# First trimester anomaly scan using virtual reality (VR FETUS study): study protocol for a randomized clinical trial

**DOI:** 10.1186/s12884-020-03180-8

**Published:** 2020-09-07

**Authors:** C. S. Pietersma, A. G. M. G. J. Mulders, L. M. Moolenaar, M. G. M. Hunink, A. H. J. Koning, S. P. Willemsen, A. T. J. I. Go, E. A. P. Steegers, M. Rousian

**Affiliations:** 1 Department of Obstetrics and Gynecology, Erasmus MC, University Medical Center Rotterdam, PO Box 2040, 3000 CA Rotterdam, the Netherlands; 2 Department of Epidemiology, Erasmus MC, University Medical Center Rotterdam, PO Box 2040, 3000 CA Rotterdam, the Netherlands; 3 Department of Radiology, Erasmus MC, University Medical Center Rotterdam, Rotterdam, the Netherlands; 4 Department of Health Policy and Management, Harvard T. H. Chan School of Public Health, Boston, USA; 5 Department of Pathology, Clinical Bioinformatics Unit, University Medical Center Rotterdam, PO Box 2040, 3000 CA Rotterdam, the Netherlands; 6 Department of Biostatistics, Erasmus MC, University Medical Center Rotterdam, PO Box 2040, 3000 CA Rotterdam, the Netherlands

**Keywords:** First trimester, Two-dimensional ultrasound, three-dimensional ultrasound, Virtual reality, Detection rate, Fetal anomalies

## Abstract

**Background:**

In recent years it has become clear that fetal anomalies can already be detected at the end of the first trimester of pregnancy by two-dimensional (2D) ultrasound. This is why increasingly in developed countries the first trimester anomaly scan is being offered as part of standard care. We have developed a Virtual Reality (VR) approach to improve the diagnostic abilities of 2D ultrasound. Three-dimensional (3D) ultrasound datasets are used in VR assessment, enabling real depth perception and unique interaction. The aim of this study is to investigate whether first trimester 3D VR ultrasound is of additional value in terms of diagnostic accuracy for the detection of fetal anomalies. Health-related quality of life, cost-effectiveness and also the perspective of both patient and ultrasonographer on the 3D VR modality will be studied.

**Methods:**

Women in the first trimester of a high risk pregnancy for a fetus with a congenital anomaly are eligible for inclusion. This is a randomized controlled trial with two intervention arms. The control group receives ‘care as usual’: a second trimester 2D advanced ultrasound examination. The intervention group will undergo an additional first trimester 2D and 3D VR ultrasound examination. Following each examination participants will fill in validated questionnaires evaluating their quality of life and healthcare related expenses. Participants’ and ultrasonographers’ perspectives on the 3D VR ultrasound will be surveyed. The primary outcome will be the detection of fetal anomalies. The additional first trimester 3D VR ultrasound examination will be compared to ‘care as usual’. Neonatal or histopathological examinations are considered the gold standard for the detection of congenital anomalies. To reach statistical significance and 80% power with a detection rate of 65% for second trimester ultrasound examination and 70% for the combined detection of first trimester 3D VR and second trimester ultrasound examination, a sample size of 2800 participants is needed.

**Discussion:**

First trimester 3D VR detection of fetal anomalies may improve patients’ quality of life through reassurance or earlier identification of malformations. Results of this study will provide policymakers and healthcare professionals with the highest level of evidence for cost-effectiveness of first trimester ultrasound using a 3D VR approach.

**Trial registration:**

Dutch Trial Registration number NTR6309, date of registration 26 January 2017.

## Background

Congenital anomalies account for 15–20% of all fetal deaths and nearly 25% of all neonatal deaths in Europe [1]. Around 2% of all pregnancies are affected by a major anomaly that requires extensive postnatal support [2]. A correct prenatal diagnosis can direct future parents to the best possible care.

According to international and Dutch national guidelines, all pregnant women are offered a second trimester two-dimensional (2D) ultrasound scan to screen for fetal anomalies [3–5]. Subsequently, future parents are counseled about the possible diagnosis, prognosis and treatment. However, by the end of the first trimester (< 14 weeks gestational age (GA)), a considerable amount of fetal anomalies can already be detected using 2D ultrasound [6, 7]. A systematic review, performed in a high risk population, showed that 61% of the (detectable) anomalies can already be identified by a first trimester anomaly scan [6]. A more recent prospective cohort study performed in The Netherlands even found a detection rate of 63% within a low risk population, which demonstrates the importance of performing a first trimester anomaly scan [8]. The observed high detection rate of major anomalies and ultrasonographic markers coincides with other advantages. Firstly, detection of an anomaly at an early stage in pregnancy provides additional time for counseling, advanced genetic testing, parental thought and reflection [9]. Secondly, a first trimester scan showing no abnormalities might provide parental reassurance, especially in a population at higher risk of fetal anomalies. Thirdly, if applicable, an early termination of pregnancy is considered a low risk procedure, in contrast to termination during advanced gestation, which shows a greater risk of maternal morbidity and mortality [10, 11]. When pregnant women opt for an early termination of pregnancy, methods available are considered less invasive when compared to termination at an advanced stage. Moreover, it has been shown that termination of pregnancy at a more advanced gestational age is associated with an increased risk of an adverse psychological outcome [12].

Finally, women with an increased BMI (i.e. > 30 kg/m^2^) could profit from a first trimester anomaly scan. It is known that the performance of the second trimester transabdominal ultrasound examination in women with an increased BMI is limited with regards to detection and completeness [13]. Incomplete ultrasound examination may lead to suboptimal care for these women. The efforts and costs greater when compared to women with a BMI < 30 kg/m^2^. In the first trimester, transvaginal ultrasound may be used to overcome limited visibility in the women with an increased BMI.

From the above mentioned, we can conclude that postponing detection of anomalies to the second trimester of pregnancy can be regarded as a missed opportunity. This is the most compelling argument mentioned in international guidelines proposing a first trimester anomaly scan to be offered to all women, accompanied by adequate counseling of the potential benefits and limitations [14]. However, there is a lack of implementation of the guidelines’ advice in nationwide screening programs. The available literature concerning detection rates and feasibility of the first trimester anomaly scan include a low risk population and are retrospective in design [6]. Prospective or randomized trials for a high risk population are limited in number. In spite of this, some countries are offering a first trimester anomaly scan to their population [15].

**Fig. 1:**
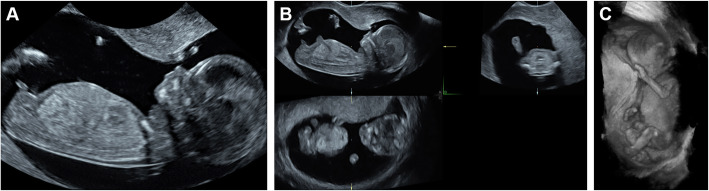
Different imaging techniques of the same pregnancy in the first trimester. Images, representing respectively transvaginal two-dimensional (panel **a**), three-dimensional (panel **b**) and three-dimensional virtual reality (Panel **c**) ultrasound images of the same pregnancy in the first trimester

Over the years several factors have contributed to the quality of first trimester 2D ultrasound imaging. The most important ones are the introduction of high frequency ultrasound probes and using the transvaginal approach. The addition of an application providing three-dimensional (3D) renderings have further enhanced first trimester ultrasound imaging by enabling improved visualization of fetal structures [16]. However, there are also limitations concerning 3D ultrasound examinations. The 3D datasets are presented as 3D reconstructions on 2D screens. Therefore, the third dimension, depth, cannot be used to its fullest since the images are being presented on 2D media. Improved visualization can be reached using Virtual Reality (VR) displays. VR enables true depth perception and provides 3D interaction. ‘Holograms’ of the 3D ultrasound scans can be created using the V-Scope software (Erasmus MC, Rotterdam, The Netherlands), allowing presentation of volumetric data in the best possible way [17, 18]. Following the acquisition of 3D data, using any 3D probe, application of V-scope imaging software enables 3D data to be displayed as ‘holograms’ on a 3D display, generating depth perception using 3D glasses. The difference between 2D, 3D and 3D VR is depicted in Fig. 1. These generated ‘holograms’, show easier to interpret images and provide detailed and relevant information about the fetal anatomy [19]. 3D VR might improve antenatal counseling of future parents.

**Fig. 2:**
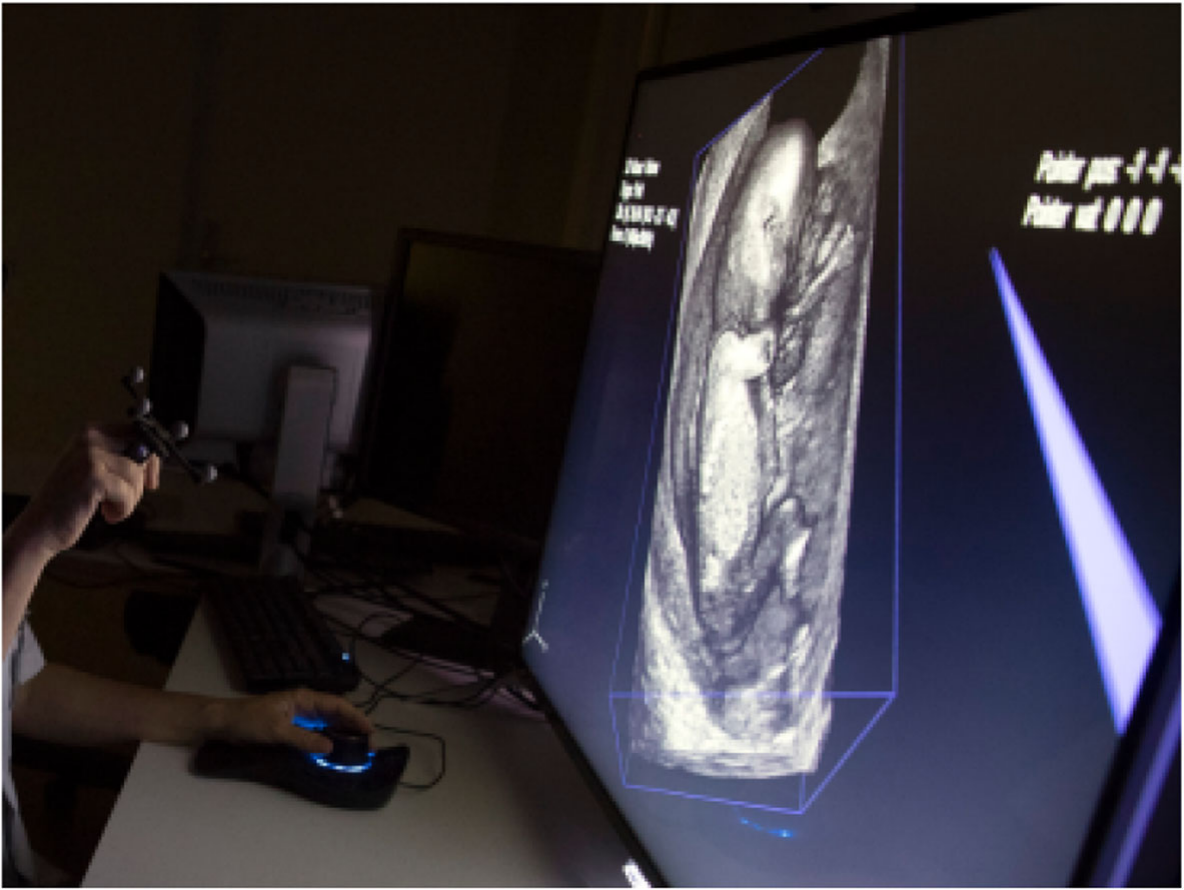
The 3D VR Desktop system in the outpatient clinic. The examiner explains the anatomical landmarks of the fetus at 13 weeks’ GA to the future parents on the Virtual Reality (VR) system. All are wearing glasses and the examiner is interacting by means of the virtual pointer. Due to privacy regulations, the examiner and future parents are not depicted in this photo. The photo was used for publication with consent of examiner and future parents

The VR technology has already been used extensively to visualize 3D ultrasound datasets in the Barco I-Space system; which is a special room enabling VR [16]. To facilitate clinical use, a 3D VR Desktop system has been developed (Fig. 2 – photo published with consent of future parents and examiner). This system can be used in daily outpatient practice, offering the same functionality at a fraction of the cost [17, 18]. Baken et al. have compared 2D ultrasound, 3D ultrasound and 3D VR for detection of first trimester fetal anomalies [20]. This retrospective study showed that the general diagnostic performance between the different methods was comparable (sensitivity 3D: 52.2%, 3D VR 62.6%; specificity 3D: 99.7%, 3D VR 99.6%). 3D VR has shown to be of additional value in the detection of anomalies, especially with regards to limb defects, conjoined twins and aneuploidy [21, 22]. Besides improving visualization, V-Scope enables the measurement of regularly used biometric features (e.g. crown-rump length) and more recently developed biometric and volumetric features (e.g. embryonic volume) with high reproducibility [23–27]. There is no difference with regards to the measurements performed in the I-Space or 3D VR Desktop system (excellent inter- and intra-observer class correlation (> 0.99)) [18].

In addition to the introduction of a first trimester anomaly scan in a high risk population, the use of VR as an additional technique is the subject of the current study. The diagnostic yield of the 3D VR will be compared with international guidelines and literature. Preferably, before implementing a new modality in daily clinical practice, cost-effectiveness should be considered [28]. Cost-effectiveness may be studied from several perspectives [29]. Ideally, this includes the societal perspective on medical costs, comprising both the direct (i.e. expenses associated with the disease and its complications) and indirect costs (e.g. effect of the illness for the patient on society, such as productivity loss). Therefore, we designed a randomized controlled trial to study the cost-effectiveness of the first trimester 3D VR ultrasound in a high risk population. Cost-effectiveness will be explored in terms of health-related quality of life and costs from a societal perspective. Also, the perspective of both patient and ultrasonographer on the new 3D VR modality will be surveyed.

## Methods/design

### Primary study objective

The general aim is to study the detection rate of a first trimester anomaly scan using 3D VR ultrasound in addition to the second trimester 2D ultrasound examination in a high risk population.

### Secondary study objectives

Additionally, we will investigate quality of life as reflected by psychological burden, and cost-effectiveness of the first trimester 3D VR ultrasound. Both factors will be compared between the intervention and control group. Finally, patient and ultrasonographer perspectives will be studied.

### Study design (see Fig. 3)

The design is a randomized controlled trial amongst women with a high risk for fetal anomalies in their pregnancy. Participants will be allocated to either the control or intervention group. In the control group, participants will receive ‘care as usual’ according to the Dutch national guidelines. The intervention group will undergo an additional first trimester 2D and 3D VR ultrasound examination. Both groups will be asked to complete questionnaires on wellbeing, quality of life, stress, anxiety and medical costs.

**Fig. 3:**
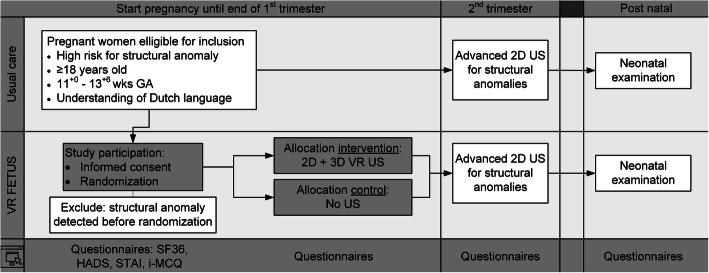
Flow chart depicting the design of the VR FETUS Study. Flowchart showing the study design of the study; including the inclusion criteria, the usual care, the intervention arm, the control arm and the follow-up. GA: Gestational Age. 2D: two dimensional. US: Ultrasound. 3D: three dimensional

### Study population

Women with an increased risk of carrying a fetus with a congenital anomaly (i.e. high risk) are eligible for participation. Women are considered high risk when: they have a previous child with an anomaly or one of the future parents has an anomaly, there is a second or third degree family member (of the fetus) with a neural tube defect, there is a maternal disease (such as diabetes or auto-immune disease), the pregnancy is conceived after IntraCytoplasmic Sperm Injection (ICSI), following teratogenic maternal medication or substance use or when there is a monochorionic twin pregnancy.

They have to meet the following additional criteria:

- 18 years of age and older
- Within the first trimester of pregnancy, up to 14 + 0 weeks’ gestation
- Singleton or twin pregnancy
- Sufficient understanding of the Dutch language

Excluded from participation are women with a non-viable pregnancy and an anomaly detected before randomization.

### Procedures, recruitment and randomization

Eligible women will be identified by the participating hospital. Referring healthcare providers will be contacted to ensure timely referral (< 14 + 0 weeks’ GA). Women eligible for the trial will be counseled before participation by medical doctors or research nurses in accordance to the ‘Good Clinical Practice (GCP)’ guidelines [30]. Written informed consent will be obtained (Supplement 1 and 2).

Following participation, the participant will be entered in a web-based computerized database (ALEA®) for randomization. After complete registration in the online randomization database, the allocation will be shown. The online randomization software is available at all times. Randomization will be performed using block randomization with random block samples of *N* = 4–16. Due to the nature of the intervention, this will not be a blinded study.

### Control group

In the control group, participants will receive ‘care as usual’ which consists of a viability and pregnancy dating ultrasound examination around 10 weeks’ gestation followed by counseling for aneuploidy testing. Aneuploidy testing will be performed either via the first trimester combined test or via non-invasive prenatal testing (NIPT). Furthermore, an advanced ultrasound examination between 18 and 22 weeks’ gestation will be performed to screen for fetal anomalies. When indicated, additional ultrasound examinations will be planned, for instance fetal cardiac evaluation with a pediatric cardiologist.

### Intervention group

The intervention consists of a first trimester 2D and 3D VR ultrasound examination between 11 and 14 weeks GA. The examination will be performed on a Voluson E10 (GE Healthcare, Austria) using a 4–9 MHz or 6–13 MHz high frequency transvaginal transducer or 2–6 MHz transabdominal transducer. A complete ultrasound examination will take 30–45 min per fetus. Ultrasound examinations will be performed according to international guidelines on safe use of ultrasound in the first trimester of pregnancy and as such, total scanning time will be kept as low as possible (ALARA-principle) [31].

The ultrasound examination will be performed by two examiners (examiner A and B) unable to see of each other’s results. Examiner A will perform the 2D ultrasound and (2D) evaluation of fetal organ structures using a protocol with standard anatomical views and measurements (Supplement 3). The cardiac examination, based on international ISUOG practice guidelines, is expanded with at least 7 measurements or planes: four chamber view with and without color Doppler, left outflow tract with color Doppler, right outflow tract with color Doppler, three vessel view, trachea view, tricuspid valve pulsed wave Doppler and a measurement of cardiac axis [32]. By default a transvaginal ultrasound examination will be performed. Only if fetal structures cannot be sufficiently visualized or if a women objects to a transvaginal examination, a transabdominal approach will be performed. During the 2D ultrasound examination, in addition to the already described 3D ultrasound datasets, four-dimensional spatio-temporal image correlation (4D STIC) datasets of the fetal heart will be acquired. Examiner B will evaluate the acquired 3D volumes using the 3D VR Desktop system [18]. Participants undergoing the first trimester scan will be unable to see the 2D scan. This is necessary to test their experience and objective reaction with respect to the 3D VR images. Thus, the influence of 3D VR ‘holograms’ on future parents’ perception can be investigated and compared to their 2D ultrasound experience later in pregnancy (at 20 weeks).

The 3D VR images will be extensively discussed with the future parents: this will take 30 min. At the end of the 3D VR evaluation and discussion, examiners A and B will compare their findings to reach agreement. In case of an anomaly, participants will be informed about the presence or suspicion of malformations by examiner B. Routine care will then be offered, consisting of an advanced anomaly scan by an experienced and independent sonographer.

In the absence of an anomaly, the intervention group will undergo a routine second trimester 2D ultrasound scan as part of ‘care as usual’.

### Virtual reality examination

The 3D VR Desktop provides a user-friendly possibility to interact with the ‘hologram’ and measure distances and semi-automatic volumes of different structures [16]. The desktop is equipped with a wireless joystick to manipulate the 3D volumes. A ‘hologram’ of the 3D volume is created using the V-Scope software (Erasmus MC, Rotterdam, The Netherlands). With stereoscopic glasses (similar to those used to watch a 3D movie) the examiners and patients (i.e. future parents) are able to perceive depth and to interact with the volume in all three dimensions. The ideal viewing angle or cutting plane can be obtained by turning, enlarging and clipping the 3D volume. The 3D VR examination will be performed using the same protocol with standard anatomical views and measurements (Supplement 3).

### Questionnaires

All participants will fill out questionnaires on health related quality of life. The following validated questionnaires will be sent at fixed time points in pregnancy: the MOS 36-item Short Form Health Survey (SF-36), the Hospital Anxiety and Depression Scale (HADS), the Spielberger State-Trait Anxiety Inventory (STAI) and a visual analogue scale (VAS) for wellbeing [33–35]. The four questionnaires will be sent digitally to the participants on the day of inclusion, the first trimester 3D VR ultrasound (or after 13 weeks GA for the control group), the second trimester 2D ultrasound and 4 weeks after the estimated due date, respectively. The answers will be recorded on a secure webserver on the Erasmus MC premises.

Direct healthcare costs will be recorded using the iMTA Medical Consumption Questionnaire (iMTA MCQ) [36]. All significant costs and health effects on the short term up to 4–6 weeks after delivery or termination of pregnancy will be considered. Therefore, these questionnaires will be sent at 20 and 32 weeks GA and 4 weeks after the estimated due date. In case of an intra-uterine fetal demise or termination of pregnancy during the trial, women will be sent specifically adjusted questionnaires.

Participant perspective on the 3D VR ultrasound will be evaluated with an in-house developed questionnaire. This survey includes questions about the value of ultrasound imaging in the first trimester and personal perception of the added value of 3D VR. This questionnaire will be sent after the first trimester 3D VR ultrasound or around 13 weeks GA in the control group. This questionnaire will be validated in a subset of 50 participants.

Ultrasonographer opinion of both the first trimester 2D and the 3D VR ultrasound will be evaluated by another in-house developed questionnaire. More specifically, the additional value of the 3D VR in terms of patient understanding, counseling and detection of anomalies will be examined.

### Follow-up

Baseline characteristics will be extracted from the electronic medical records and stored in a secured database. Only a select group of staff will have access to participant characteristics to ensure patient confidentiality.

Hospital or midwifery charts will be obtained to complete follow-up on neonates. In case of a fetal anomaly, additional information will be obtained, such as genetic testing or autopsy results.

### Outcome measurements

The primary outcome will be the detection rate of fetal anomalies using first trimester 3D VR ultrasound in addition to the second trimester 2D ultrasound in a high risk population. Neonatal or histopathological examinations are considered the gold standard for the detection of congenital anomalies. For the detection rate analysis the sensitivity, specificity, positive and negative predictive values will be calculated for all ultrasound techniques for first trimester examination (i.e. 2D and 3D VR) in the intervention group and second trimester 2D examination in both groups (control group as well as intervention group). The detection rate of both first trimester examinations (i.e. 2D and 3D VR) will be compared. The detection rate of first trimester 2D will also be compared to the second trimester 2D ultrasound in the intervention group. In addition, we will assess the performance of the first trimester anomaly 2D and 3D VR ultrasound and the additional findings of the subsequent second trimester 2D ultrasound to gain a better insight into the whole diagnostic process.

The diagnostic performance of the first trimester ultrasound examination protocol will be compared to international ultrasound guidelines. Anatomical views (i.e. sagittal, transversal) according to our standardized protocol acquired per fetal structure will be evaluated as satisfactory or unsatisfactory. This assessment will be used to evaluate image quality per fetal structure and per modality, i.e. first trimester 2D and 3D VR. By doing this we will be able to examine the anatomical views which are essential to be included in the protocol, and the ones which are not. This evaluation will lead to optimization of the examination protocol in the future.

Secondary outcome measures will be health-related quality of life and cost-effectiveness of first trimester 3D VR ultrasound from a societal perspective. Health related quality of life will be expressed in physical, emotional and social functioning. Quality Adjusted Life Years (QALY’s) will be calculated from a maternal perspective, derived from the validated questionnaires. Actual expenses during the study will be measured using the standard costs as stated by the Dutch costing guidelines [37]. For each strategy (control versus intervention) the quality of life will be computed as well as the average costs per patient. The incremental cost-effectiveness ratio (ICER) will be calculated by dividing the difference in costs by the difference in quality of life. It represents the extra costs required to obtain one QALY. Furthermore, the cost per anomaly detected will be estimated. The cost-effectiveness analysis will span a period from approximately 8 weeks of gestation, when regular obstetric care starts, up to approximately 2 months beyond the estimated date of delivery. In this analysis, costs and effects will not be discounted due to a short time frame. Indirect costs will not be accounted for. The costs will be presented in Euros. The index year will be 2018.

Additionally, through a newly implemented questionnaire, the perspective of both patient and ultrasonographers on the new 3D VR modality will be surveyed. The participant perspective of the first trimester 3D VR ultrasound group (=intervention) will be compared to second trimester 2D ultrasound (=control).

An overview of all patients’ characteristics and data is shown in Table 1.

**Table 1:** Patient characteristics collected within the VR FETUS study. Details of data collection form the first trimester up to 6 weeks after delivery are depicted

**Maternal Characteristics**	**Outcome**
Age	Years (mean ± SD)
Ethnicity	Dutch/Other western/Non-western
Level of education	Low, middle, high
Postal code	
Medical history	Unremarkable/Diabetes/Epilepsy/Other
Obstetrical history	Parity, miscarriage, previous pregnancy with congenital anomaly
Family history	Unremarkable/family members with congenital anomaly
Marital status	Married/not married
Global household income	€0–20.000/€20.000–35.000/€35.000–65.000/>€65.000 annually
BMI	Kg/m^2^ (mean ± SD)
Alcohol use during first trimester	No/Yes: how many units per day?
Smoking during first trimester	No/yes: how many cigarettes per day
Recreational drug use during first trimester	No/yes: what drugs; frequency per day?
Folic acid use during and/or prior to pregnancy	No/yes during pregnancy/yes started prior to pregnancy
Pregnant after artificial reproductive technology	No/yes: what type of assisted reproduction
First trimester screening for aneuploidy	No/yes: result
Medication	No/yes: what medication, dosage, starting and stopping date?
Antidepressant	Never/yes currently/yes previous use
**Questionnaires**	**Timing**
Current health status^a^	At inclusion, intervention or 13 weeks’ GA, 20 weeks’ GA, 6 weeks after due date
Anxiety and depression^b^	At inclusion, intervention or 13 weeks’ GA, 20 weeks’ GA, 6 weeks after due date
State and trait anxiety^c^	At inclusion, intervention or 13 weeks’ GA, 20 weeks’ GA, 6 weeks after due date
Current health status by VAS^d^	At inclusion, intervention or 13 weeks’ GA, 20 weeks’ GA, 6 weeks after due date
Medical consumption^e^	At 20 weeks’ GA, 32 weeks’ GA, 6 weeks after due date
Patient satisfaction with 3D VR	At intervention or 13 weeks’ GA, 6 weeks after due date
**Ultrasound**	**Measurements**
At inclusion, 1st trimester	Viability, CRL, multiple pregnancies, location
Intervention: 2D + 3D VR First Trimester Ultrasound, 1st trimester	Fetal anomaly scan using 2D and 3D VR. Growth parameters, detection of anomaly. See supplement A
Advanced fetal anomaly scan, 2nd trimester	Fetal anomaly scan using 2D. Growth parameters, detection of anomaly
**Pregnancy outcome**	**Outcome**
Outcome	Live birth, termination, intra-uterine fetal demise
Mode of delivery	Vaginal / Instrumental delivery / Cesarean section
Gestational age at delivery	Days (mean ± SD)
Invasive testing	No/yes: result
Date of invasive testing	
**Neonatal outcome**	**Outcome**
Birthweight	Birthweight, grams (mean ± SD)
Gender	Male/female
Congenital malformation	No/yes: what malformation
Prenatal diagnosis	Confirmed/discrepancy in findings: postnatal new or other findings

^a^SF-36: MOS 36-item Short Form Health Survey, ^b^HADS: Hospital Anxiety and Depression Scale, ^c^STAI: Spielberger State-Trait Anxiety Inventory, ^d^VAS: visual analogue scale, ^e^iMTA MCQ: iMTA Medical Consumption Questionnaire, CRL: Crown-Rump Length, 2D: two-dimensional, 3D VR: three-dimensional virtual reality

### Quality

Sonographers will be trained to perform first trimester 3D VR ultrasound scans. As the first trimester ultrasound scan for fetal anomalies is currently not part of standard care in the Netherlands, the sonographers will receive additional training. They will all collect a portfolio comprising 5 complete ultrasound examinations, which will be reviewed by two experts. Unsatisfactory portfolios will be discussed with the sonographers in order to improve quality. When at least 5 high quality examinations of different patients have been collected, the sonographer can participate in the study.

To ensure quality of the first trimester ultrasound, all sonographers are asked to compile a new portfolio every 200 first trimester ultrasound scans.

### Statistical issues

#### Sample size

The prevalence of anomalies in the high risk group is 5–10% [38, 39]. The detection rate for fetal anomalies with 2D ultrasound in the first trimester lies between 18.2 and 71.8% [7]. As mentioned before, the detection rate in a high risk population is estimated at 61%, and in a low risk population at 63% for 2D ultrasonography [6, 8]. The detection rate for anomalies of the first trimester 3D VR ultrasound is 62.5% [20]. For second trimester (18–22 weeks’ gestation) 2D ultrasound the detection rate lies between 44.3 and 74.4% [40–42].

For sample size calculation with a superiority analysis we assumed a 65% detection rate for a second trimester 2D ultrasound examination and a 70% detection rate for the combined detection of first trimester 3D VR and second trimester 2D ultrasound examination in a high risk population. With an assumed prevalence of 7.5% and an alpha-error of 0.05 with a desired power of 0.80, a total group of *N* = 2800 is required.

#### Data-analysis

Data will be analyzed according to the intention-to-treat principle. No interim analysis will be performed.

Statistical analysis will be performed using IBM SPSS (SPSS Inc., Chicago, IL, USA) and R Studio (The R Foundation for Statistical Computing). Descriptive statistics will be used to describe the baseline characteristics of both the control and intervention group (e.g. maternal characteristics such as ethnicity, age, smoking status, Body-Mass Index (BMI) and family history, pregnancy characteristics such as parity and pregnancy outcome, medication use such as antidepressants and antiepileptic medication). Categorical data will be presented by number of participants (%) and numerical data by median (interquartile range). These baseline characteristics will be compared between the arms using Mann Whitney U-test for continuous variables and chi-squared test for categorical variables. When the expected count in 20% or more of the cells of the cross table for the categorical variables is 5 or lower or if any cell is empty an exact test will be used instead. A significance level (alpha) of 0.05 will be used. No multiplicity correction will be applied.

The economic analysis will be performed from the societal perspective. The costs and quality of life will be compared between the two diagnostic regimens. Descriptive statistics will be used to describe the outcomes of quality of life analysis (control arm versus intervention arm).

Ultrasound is considered safe in pregnancy, therefore a data monitoring committee is not required [43]. Yearly reports will be sent to the ethics committee on the progress of the trial. When major modifications to the protocol are made, all people involved will be informed.

## Discussion

This is the first randomized controlled trial designed to detect anomalies in the first trimester using 3D VR ultrasonography, with longitudinal follow-up in pregnancy. One previous study has shown a high sensitivity for the detection of structural anomalies in the first trimester using 3D VR and a trend towards improved detection rates [16]. 3D VR may especially improve the detection of surface anomalies in the first trimester [20]. Timely detection of major congenital malformations will provide women at risk with additional time to perform (genetic) testing as well as more time to consider continuation or termination of pregnancy. In current Dutch practice even women from the high risk population are only offered a second trimester anomaly scan. In the high risk group an additional first trimester ultrasound examination may be beneficial since there will be an increased prevalence of anomalies. Furthermore, the absence of a major anomaly following a first trimester 3D VR ultrasound examination could offer earlier reassurance and reduce anxiety in pregnant women. Overall, we hypothesize this will improve cost-effectiveness and patient quality of life.

This study has several strengths. The use of a standardized examination protocol will improve the detection of fetal anomalies. Recent studies have shown increased detection rate of anomalies when a standardized ultrasound protocol is used compared to a strategy that consists of a global fetal survey [6]. Also, transvaginal ultrasound or the combination of transvaginal and transabdominal ultrasound may increase the detection of anomalies in the first trimester compared to only using a transabdominal approach. This is especially true in case of maternal obesity. Detection of anomalies will be further improved by the limited filtering of the ultrasound data by the 3D VR system, which can enhance fetal surface details and thus improve the examination of limbs and face. This technique also provides recognizable and identifiable ‘holograms’ for the future parents.

Some challenges remain. The introduction of a first trimester ultrasound examination will necessitate an additional prenatal visit, as well as expertise and training of staff. This might increase healthcare costs. This study will add new understanding on maternal psychological functioning throughout pregnancy in relation to prenatal ultrasound. Ideally, we would use structured interviews. However, due to the size of the study this was not a possibility. Instead we chose to use validated questionnaires to investigate maternal stress and anxiety.

Some organ systems are not yet fully developed in the first trimester or are too small to be visualized in detail. As an example, one can state that it is not possible to detect all or a large proportion of fetal cardiac and brain anomalies in early pregnancy. Therefore, second trimester ultrasound examination cannot be omitted, and has to be used in addition to first trimester ultrasound.

From a clinician’s point of view, the first trimester ultrasound should be studied regarding its additional value of diagnostic performance. Rather than comparing first and second trimester ultrasound examination, the performance should be based on the combination of first and second trimester. We expect that first trimester detection will show an improvement in the clinical care of participants and lead to significant improvement in their quality of life. Furthermore, a second trimester ultrasound examination will show the development of a congenital anomaly over time. Parents’ decision for continuation of the pregnancy is likely to be affected by this. The second trimester ultrasound is indispensable, therefore we will study the combined first and second trimester ultrasound examination compared to the second trimester only.

This study will provide the highest level of evidence in the evaluation of first trimester 3D VR ultrasonograhpy in a high risk population concerning quality of life and cost-effectiveness. Only with a randomized controlled trial, like this study, the additional value of the 3D VR desktop can be examined in terms of improved diagnostic yield of regular first trimester 2D ultrasound and patients perspectives. If this new modality proves to be cost-effective or shows to improve quality of life, it should be offered in addition to the second trimester 2D ultrasound examination. The wider implications of the results will give policymakers and healthcare professionals an educated choice as whether to add first trimester ultrasound for detection of congenital anomalies in a high risk group as part of routine care.

## Supplementary information

**Additional file 1.** Patient information.

**Additional file 2.** Information for future parents.

**Additional file 3.** Checklist structural ultrasound examination 1st trimester.

## Data Availability

Not applicable. The trial data will not become publicly available but will be available upon reasonable request to the corresponding author.

## Abbreviations

3D: Three-dimensional
2D: Two-dimensional
VR: Virtual Reality
STAI: Spielberger State-Trait Anxiety Inventory
HADS: Hospital Anxiety and Depression Scale
SF36: MOS 36-item Short Form Health Survey
iMTA MCQ: Institute for Medical Technology Assessment Medical Consumption Questionnaire
GA: Gestational Age
NIPT: Non-Invasive Prenatal Testing
QALY: Quality Adjusted Life Years
ICER: Incremental cost-effectiveness ratio
BMI: Body-Mass Index
