# Supplementary material for: First trimester anomaly scan using virtual reality (VR FETUS study): study protocol for a randomized clinical trial
Source: BMC Pregnancy Childbirth. 2020 Sep 7;20:515. doi: 10.1186/s12884-020-03180-8 (PMC7487721; doi:10.1186/s12884-020-03180-8)
Supplement: Supplementary file 1 — Additional file 1. Patient information. [file 12884_2020_3180_MOESM1_ESM.docx]

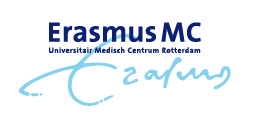
***Patient information***

***"The additional value of first trimester three-dimensional virtual reality ultrasound scan in obstetric care: a randomized study"***

***"The usefulness of three-dimensional virtual reality ultrasound during the first three months of pregnancy”***

Dear Madam,


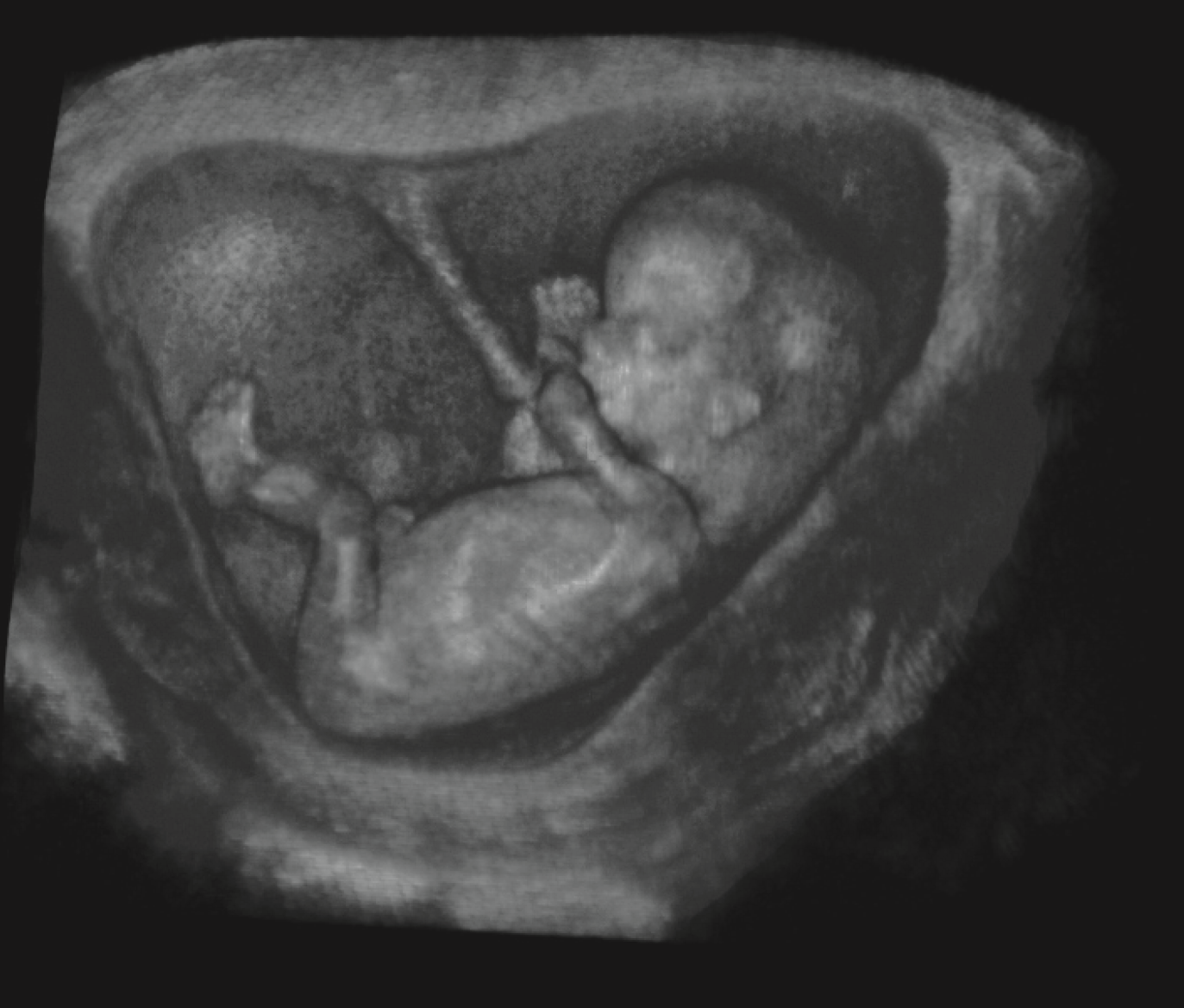
We would like to ask you to participate in a medical scientific study. During this study, we will investigate whether three-dimensional virtual reality (VR) ultrasound, a technique that can display depth, during the first three months of pregnancy can detect birth defects in your child. Before making the decision to participate in this study, it is important to know more about this research. Please read this information letter, after which you can discuss it with your partner, friends or family. There is also a confidential advisor appointed not involved in this study, but otherwise well informed, that you can consult (contact details can be found on page 8). Attached you will also find a general brochure about medical scientific research. If you have any questions after reading the leaflet, you can consult the research team. The contact details can be found on page 7.

You are a patient at the Erasmus MC Department of Fetal Medicine, because unfortunately your child has an increased risk of a birth defect. During pregnancy, women often get one or more ultrasounds (this is also called an ultrasound examination). During an ultrasound examination, one can determine, amongst other, how far you are pregnant and whether there are any abnormalities in your baby. An ultrasound investigation is an imaging examination that uses ultrasound waves. The examination itself is not painful. A standard ultrasound examination is a two-dimensional (2D) examination. This means that the image that is recorded is always a cross-section (a single plane) of the child.

During your pregnancy, you are eligible for an extensive ultrasound examination that takes place between 18 and 22 weeks of gestation. During this examination, we systematically check your child and assess whether there is an anomaly present. This research is called the *Advanced Ultrasound Examination.*

A three-dimensional (3D) imaging technique that we can use performing an ultrasound examination has recently been developed at the Erasmus MC, University Medical Center in Rotterdam. Using specific software true depth can be displayed with this 3D VR technique. Hence, this 3D VR (VR) technique assists the ultrasound operator's assessment of standard 2D ultrasound images. The 3D VR technique also allows the woman and her partner to view the images. We would like to ask you if you would like to participate in scientific research into this 3D VR technique.

This research is performed at the Erasmus MC, University Medical Center in Rotterdam in collaboration with hospitals in the region. In four years 2,800 women will participate who have an increased risk of having a child with a birth defect.

**1. What is the purpose of the study?**

Recent research shows that standard ultrasound examination for birth defects can be performed during the 13 week ultrasound scan (with a gestational age of 11-13 weeks) instead of during the 20 week ultrasound scan (with a gestational age of 18 - 22 weeks). This is possible due to the increased knowledge about birth defects, the increased experience of the sonographer and new equipment.

During this research, we want to study whether we can use the recently developed 3D VR technique to diagnose birth defects in your child earlier in pregnancy. With questionnaires we want to investigate at the influence of the 3D VR technique on patients and healthcare providers.

**2. Which diagnostic test is being investigated?**

During this study, one half of the participating pregnant women will have a 13-week ultrasound with the 3D VR technique (i.e. during the first three months of pregnancy). With the help of ultrasound equipment, both a 2D and a 3D image are made, which is processed with special software. The image can then be viewed on a special screen. Depth can be observed with the help of this 3D screen. You can compare this with a 3D film: if you watch without 3D glasses, you do not get a clear picture. When you put on the 3D glasses, you can perceive depth.

**3. How is this study conducted?**

In this study, we want to investigate whether we can gather important information about the presence or absence of a birth defect using the 3D VR technique at an early stage of pregnancy. To be able to assess whether the 3D VR 13 weeks ultrasound scan (first trimester of pregnancy) is better than the extensive 2D 20 week ultrasound scan (second trimester of pregnancy), which is offered to all pregnant women at increased risk of having a baby with a birth defect, the participants are divided into two groups. One group receives the standard extensive ultrasound examination around a pregnancy duration of 20 weeks (the 20 2D week ultrasound scan). The other group will receive an extra ultrasound in the Erasmus MC, the 3D VR 13 week ultrasound scab, during the first three months of pregnancy. This group is then also offered the standard extensive ultrasound scan around a gestational age of 20 weeks.

To ensure that the distribution between the two groups is as correct as possible, the distribution is determined by drawing lots. That draw is called randomization. Your treating physician and the researchers have no influence on the result of the draw. So you have a 50% chance that you will be assigned to the group that will receive an extra ultrasound scan (the 3D VR ultrasound).

The study will take a total of 4 years. If you go through the entire study period, this will take you from the period that you are 11-13 weeks pregnant to the first check-up after pregnancy. This initial checkup often takes place within 6 weeks of the baby's birth.

If you decide to participate and you draw for the group with a standard treatment, you will **not** receive a 3D VR 13 week ultrasound. You will receive as part of standard of care an ultrasound scan to determine the gestational age. You will then receive the extended 2D 20- week ultrasound scan at 18-22 weeks. This scan is offered to all women with an increased risk of having a child with a birth defect and is not part of this study. If there is a medical reason, the ultrasound examination is repeated at 30 weeks of gestation. This ultrasound scan also concerns standard care. When you are placed in the group with standard treatment, no extra ultrasounds are made than is custom in standard care. If a medical reason arises during pregnancy, ultrasound examinations during pregnancy are carried out more frequently. This can also be done in the hospital where the usual antenatal care takes place.

If you are placed in the group of the 3D VR 13 week ultrasound scan, one extra visit will be scheduled. During this extra visit, you will receive an extensive 2D 13- week ultrasound scan. A researcher (researcher A) also makes 3D recordings. You should take into account that the 3D VR 13 week ultrasound will be performed transvaginaly. After the researcher has finished the ultrasound scan, the 3D VR images will be assessed by another researcher (researcher B). Together with you researcher B will then go through the 3D images and discuss the results. The entire visit is expected to last approximately 1 hour.

When an abnormality is discovered at the 13 week ultrasound, you may need another ultrasound examination. This is standard concern if abnormalities are seen on ultrasound examination. You will receive an explanation and advices if birth defects are found. For some of the birth defects found with the 13 week ultrasound, the consequences for the child's future health are unknown.

In addition to the ultrasound examinations, during the study participation questionnaires are also distributed. With the help of the questionnaires, we obtain information about your feelings (fear / anxiety / perception of your quality of life) and about your experience with the ultrasound examinations. You will also receive a questionnaire in which we will ask you to keep track of costs you incur during the period of participation in the study. In total, you will be asked to fill in up to 24 questionnaires during the study. These take about 10 minutes to fill in. The women who undergo the 3D VR 13 week ultrasound are asked to complete two additional questionnaires compared to the group that only undergoes the standard ultrasound scan. In addition, some general information about you and the pregnancy will be collected, including your age, ethnicity and education level. Finally, permission is requested to request general and medical data in order to be able to compare the baby's data with the ultrasound images.

**4. What is expected of you?**

If you decide to participate in the study, we ask you to honor the agreements made and to follow the instructions as closely as possible. What participation in the study specifically means, can be found elsewhere in this information letter.

**5. What is extra or different from the regular treatment(s) you receive?**

The ultrasounds that are covered by standard care can be found in the introduction to this information letter. If you participate in the study, it is the intention that, when you are allocated to the 3D VR group, one extra ultrasound examination (2D 13 week ultrasound and 3D VR 13 week ultrasound) will be performed during the first three months of the pregnancy. These ultrasound examinations will be performed immediately one after the other. So you only have to visit the hospital one time extra. If a birth defect is seen, you may have to come again shortly after the 13-week ultrasound for an extra ultrasound, which is part of standard care. In appendix 4, a table of all the ultrasound visits and questionnaires is shown. Completing the questionnaires is extra effort for both groups. In the table, it is also indicated which investigations are part of standard care and which are performed extra for the study.

**6. What side effects can you expect?**

Participation in the study does not pose a risk to you or your child.

**7. What are the possible advantages and disadvantages of participating in the study?**

In most cases, the 3D VR 13 week ultrasound will provide reassurance. In about 5-10 % of the pregnancies a (possible) birth defect will be visible. When a birth defect is detected early in the pregnancy, you have more time to think about doing additional tests. You decide which additional tests you want to have or do not want to have. You therefore have more time to think about the future possibilities. With some findings, it is not immediately clear whether this is normal or abnormal: this can cause uncertainty. The finding will therefore be monitored over time with additional ultrasound examination(s).

In principle, the 13-week ultrasound is a transvaginal ultrasound. Discomfort from transvaginal ultrasound may be experienced.

Furthermore, we ask you to fill in up to 24 questionnaires. The questions may be personal and/or confrontational. The time required for answering the questionnaires can also be burdensome. It takes about 10 minutes to complete one round of questionnaires.

**8. What happens if you do not wish to participate in the study?**

You decide whether you want to participate in the study. Participation is voluntary. If you decide not to participate, you do not need to do anything else. You do not have to sign anything. You also do not have to say why you do not want to participate. You simply receive the treatment that you would have received otherwise. If you do participate, you can always change your mind and still stop your participation. Also during the study.

**9. What happens when the study is finished?**

Since the ultrasounds are performed as part of study, you will not receive your personal results. Any findings that are of interest to you personally will be discussed with you by the researcher. If you do not want this, you cannot participate in this study.

**10. Will you have a subject insurance when you participate in the study?**

Human subject insurance is provided for everyone participating in the study. The subject insurance covers damage resulting from the study. This applies to damage that emerges during the study or within four years of the end of the study. Appendix 3 takes you to the insured amounts, exceptions and details of the insurance company.

**11. Will you be informed if relevant information about the study becomes available for you in the meantime?**

The study will proceed as accurately as possible. If unexpected findings are found for you or your child during the examination, you will be informed and, if necessary, you will be referred to a medical specialist. If you do not want this, you cannot participate in this study.

**12. How are your data used and stored?**

Your personal data is collected, used and stored for this study. This concerns information such as your name, address, ethnicity, date of birth and information about your health. The assembly, use and storage of your data is necessary to answer the questions asked in this study and to publish the results. We ask your permission for the usage of your data.

**Confidentiality of your data**

Your data is given a code to protect your privacy. Your name and other data that can directly identify you are anonymized. Tracing the data back to you is only possible with key of this code. The key of the code remains safely stored in the local research institution. The data sent to the researcher only contains the code, but not your name or other data that can identify you. The data cannot be traced back to you in reports and publications about the research.

**Access your data for verification**

Some persons may access your data at the research site. Also to the data without code. This is necessary in order to check whether the research has been carried out properly and reliably. Persons who have access to your data for inspection are: the committee that monitors the safety of the study, a monitor that works for the sponsor of the investigation, national and international supervisory authorities (for example, the Health and Youth Care Inspectorate). They will keep your data secret. We ask you to give permission for this access.

**Data retention period**

Your data must be stored at the research location for 15 years after the end of the study.

**Retention and use of data for other research**

After this study, your data may also be important for other scientific research in the area of ​​the further development of the ultrasound method. For this, your data will be stored 15 years after the end of the study. You can indicate on the consent form whether you agree to this.

**Information about unexpected findings**

During this examination, by chance something may be found that is not of interest to the examination but is of interest to you. If this is important for your or your child’s health, you will be informed by the doctor or midwife of the Department of Fetal Medicine. You can then discuss with your midwife or specialist what additional tests needs to be done. You also give permission for this.

**Revoke permission**

You can always withdraw your permission to use your personal data. This applies to this study and to the storage and use for future research. The research data collected until your withdrawal of consent will still be used in the study.

**More information about your rights when processing data**

For general information about your rights when processing your personal data, you can consult the website of the Dutch Data Protection Authority.

If you have any questions about your rights, please contact the person responsible for the processing of your personal data. For this study, it is Erasmus MC. See appendix 1 for contact details.

If you have any questions or complaints about the processing of your personal data, we recommend that you first contact the research location. You can also contact the Data Protection Officer of the institution, see appendix 1 for contact details. You can also contact the Dutch Data Protection Authority.

**Registration of the study**

Information about this research is also included in an overview of medical scientific studies, namely the Dutch Trial Register. It does not contain any data that can be traced back to you. After the study is finished, the website may show a summary of the results of this investigation. You will find this study under ‘Virtual Reality & Feasibility and Efficacy of first Trimester Ultrasound: a randomized controlled trial'.

**13. Are there additional costs / is there a fee if you decide to participate in this study?**

There are no additional costs for you to participate in the study. For each exam, ultrasound images of the exam are printed on paper for you to take with you. You will not be reimbursed for travel expenses incurred.

The researcher and the institution are paid for your participation in the study. However, our researchers do not personally benefit from your participation in this study. Such a fee is used to support the research program.

**14. Which medical ethics review committee has approved this study?**

The Erasmus MC Medical Ethical Review Committee has approved this study. More information about the approval can be found in the General brochure.

**15. Do you want to know anything else?**

If you have any questions or complaints during the investigation, we ask you to contact your treating physician or an employee of the study team: see appendix 1 for contact details.

If you are unsure about participating, you can consult an independent doctor who is not involved in the study itself, but who is an expert in the field of this study. If you have questions, that you do not dare to propose to the investigators, before or during the investigation, you can contact the independent physician.

If you are not satisfied with the examination or treatment, you can contact the independent complaints office of Erasmus MC.

If, after careful consideration, you have decided to take part in this scientific study, we ask you to sign and date the consent form together with the researcher.

Sincerely,

The study team

Dr. A.G.M.G.J. Mulders, gynecologist-obstetrician, Erasmus MC

Dr. M. Rousian, gynecologist in training, Erasmus MC

Drs. C.S. Pietersma, physician at the Fetal Medicine Department, Erasmus MC

Attachments

- Appendix 1: Contact details

- Appendix 2: General information letter regarding scientific research with humans

- Appendix 3: Subject insurance information

- Appendix 4: Overview of extra ultrasound visits and questionnaires

**Appendix 1: Contact details**

Lead researcher:

Dr. A.G.M.G.J. Mulders, gynecologist-obstetrician tel. +3110 - 703 3492

Co-researcher:

Dr. M. Rousian, gynecologist in training tel. +3110 - 703 3492

Drs. C.S. Pietersma, physician Fetal Medicine tel. +316 - 2824 6981

Independent doctor:

Prof. dr. I.K.M. Reiss, neonatologist tel. +3110 - 703 60 77

Complaints Office of Erasmus MC University Medical Center:

tel. +3110 - 703 31 98

Data protection officer:

The Erasmus MC Data Protection Officer can be reached through the secretariat of the Legal Affairs Department: tel. +3110 - 703 49 86

Website of Dutch Data Protection Authority:

www.autoriteitpersoonsgegevens.nl

Website Dutch Trial Register – Virtual Reality FETUS study

<http://www.trialregister.nl/trialreg/admin/rctview.asp?TC=6309>

**Appendix 2: General information letter regarding scientific research with humans**

This can also be viewed at the website of the Dutch government: <https://www.government.nl/topics/medical-research>

**Appendix 3: subject insurance information**

The sponsor has arranged subject insurance for everyone who participates in this study. The insurance covers damage due to participation in the study. This applies to damage during the investigation or within four years after its end. You must report damage to the insurer within those four years.

The insurance does not cover all damage. At the bottom of this text is shown briefly what damage is not covered. These provisions are set out in the Decree on compulsory insurance for medical research involving human subjects. This decision can be found on www.ccmo.nl, the website of the Central Commission for Human Research (see ‘Human subjects’ and then 'Rights and obligations').

In the event of damage, you can contact the insurance company directly:

Name: CNA Insurance Company Limited

Address: Polarisavenue 140

2134 JX Hoofddorp

Contact agent: Ms. Esther van Herk

Tel: +3120 – 303 60 04

Email: [esther.vanherk@cnaeurope.com](mailto:esther.vanherk@cnaeurope.com)

The insurance provides cover of € 650,000 per subject and € 5,000,000 for the entire study and € 7,500,000 per year for all studies from the same client.

The subject insurance does not cover the following damage (this is subject to change, typing errors and translation errors and therefore has no legal value):

- damage due to a risk about which you have been informed in the written information. This does not apply if the risk is more serious than anticipated or if the risk was very unlikely;

- damage to your health that would have occurred even if you had not participated in the study;

- damage caused by not (fully) following directions or instructions;

- damage to your offspring, as a result of a negative effect of the research on you or your offspring;

- damage due to an existing treatment method when investigating existing treatment methods.

**Appendix 4: overview of extra ultrasound visits and questionnaires**

Regular visits, extra visits and questionnaires during this study.

|  | **Pregnancy before 11-13 weeks’ gestation** | **Pregnancy between 11-13 weeks’ gestation** | **Pregnancy at 16 weeks’ gestation** | **Pregnancy at 18-22 weeks’ gestation** | **Pregnancy at 30 weeks’ gestation** | **6 weeks after delivery** |
| --- | --- | --- | --- | --- | --- | --- |
| **Screening and information (5 minutes)** | x  (standard care) |  |  |  |  |  |
| **Signing and dating consent forms** | x |  |  |  |  |  |
| **Answer questionnaires (maximum 10 minutes per questionnaire)** | x  (4 pieces) | x  (5 pieces) | x  (4 pieces) | x  (5 pieces) | x  (1 piece) | x  (6 pieces) |
| **Request general and medical data, when permission is given** | x  (standard care) |  |  |  |  | x  (standard care) |
| **Ultrasound examination** | Dating ultrasound (standard care) | 3D VR ultrasound at the Erasmus MC (only for those in the 3D VR group) | 2D ultrasound (only with a medical indication and for those in the 3D VR group) | 2D ultrasound (standard care) | Only with medical indication |  |

**Consent form expectant mother (copy patient)**

**"The added value of first trimester three-dimensional virtual reality ultrasound scan in obstetric care: a randomized study"**

**"The use of three-dimensional virtual reality ultrasound during the first three months of pregnancy”**

Study number: 

I confirm that I have read the information brochure about the study and understand the information. I have had the opportunity to ask additional questions. These questions were answered satisfactorily. I have had enough time to think about participating.

I know that my participation is completely voluntary and that I can withdraw my consent at any time without motivation.

I authorize the viewing and retrieval of my medical and research data by authorized employees of the research team, employees of the Health Care Inspectorate, members of the Medical Ethics Committee Erasmus MC and persons appointed by the researchers to check and aid the study.

I give permission to use my data for the purposes as described in the information brochure.

I authorize the storage of my research data and ultrasound images 15 years after the end of this research.

I declare to participate voluntary in this medical scientific research using power Doppler and 3D ultrasound during pregnancy.

|  | **Yes** | **No** |
| --- | --- | --- |
| I give permission to inform my midwife, general practitioner and / or medical specialist, who is treating me, that I am participating in this study and to inform them of the ultrasound findings. | □ | □ |
| I give permission to use my data and ultrasound images for a maximum of 15 years after this study for future studies into the possibilities of virtual reality ultrasound to discover whether there are signs of birth defects or studies that examine the development of the child and detecting birth defects. | □ | □ |

|  | **Yes** | **No** |
| --- | --- | --- |
| I give permission of the use of ultrasound, Doppler, MRI, video or other images for possible publication†, provided that these images are anonymous and therefore cannot be traced back to my child or me.  *^†^Publications include: journal, abstract, brochure, poster, presentation, teaching materials for a course or clinical class, or PR.* | □ | □ |
| I give permission to contact me in the future for follow-up studies | □ | □ |
| I give permission to request information about me from the Municipality / Statistics Netherlands (they have information on the family composition, whether you have moved and unexpected mortality has occurred). | □ | □ |
| I give permission to request information about me from my pharmacy. | □ | □ |
| I give permission to request information about me from the FARMA Foundation, the Pharmacological Key Figures Foundation (which store data on the use of medicines). | □ | □ |

I want to participate in this study.

| **Expectant mother** |  |
| --- | --- |
| Name:  Date:  Signature: | …………………………………………………..  _ _ - _ _ - 2 0 _ _  ………………………………………………….. |

I hereby declare that I have fully informed this subject about the above-mentioned study. If during the research, information becomes available that could influence the consent of the subject, I will inform her in due time.

| **Researcher (or his representative)** |  |
| --- | --- |
| Name:  Date:  Signature: | …………………………………………………..  _ _ - _ _ - 2 0 _ _  ………………………………………………….. |

**Consent form expectant mother (copy researcher)**

**"The added value of first trimester three-dimensional virtual reality ultrasound scan in obstetric care: a randomized study"**

**"The use of three-dimensional virtual reality ultrasound during the first three months of pregnancy”**

Study number: 

I confirm that I have read the information brochure about the study and understand the information. I have had the opportunity to ask additional questions. These questions were answered satisfactorily. I have had enough time to think about participating.

I know that my participation is completely voluntary and that I can withdraw my consent at any time without motivation.

I authorize the viewing and retrieval of my medical and research data by authorized employees of the research team, employees of the Health Care Inspectorate, members of the Medical Ethics Committee Erasmus MC and persons appointed by the researchers to check and aid the study.

I give permission to use my data for the purposes as described in the information brochure.

I authorize the storage of my research data and ultrasound images 15 years after the end of this research.

I declare to participate voluntary in this medical scientific research using power Doppler and 3D ultrasound during pregnancy.

|  | **Yes** | **No** |
| --- | --- | --- |
| I give permission to inform my midwife, general practitioner and / or medical specialist, who is treating me, that I am participating in this study and to inform them of the ultrasound findings. | □ | □ |
| I give permission to use my data and ultrasound images for a maximum of 15 years after this study for future studies into the possibilities of virtual reality ultrasound to discover whether there are signs of birth defects or studies that examine the development of the child and detecting birth defects. | □ | □ |

|  | **Yes** | **No** |
| --- | --- | --- |
| I give permission of the use of ultrasound, Doppler, MRI, video or other images for possible publication†, provided that these images are anonymous and therefore cannot be traced back to my child or me.  *^†^Publications include: journal, abstract, brochure, poster, presentation, teaching materials for a course or clinical class, or PR.* | □ | □ |
| I give permission to contact me in the future for follow-up studies | □ | □ |
| I give permission to request information about me from the Municipality / Statistics Netherlands (they have information on the family composition, whether you have moved and unexpected mortality has occurred). | □ | □ |
| I give permission to request information about me from my pharmacy. | □ | □ |
| I give permission to request information about me from the FARMA Foundation, the Pharmacological Key Figures Foundation (which store data on the use of medicines). | □ | □ |

I want to participate in this study.

| **Expectant mother** |  |
| --- | --- |
| Name:  Date:  Signature: | …………………………………………………..  _ _ - _ _ - 2 0 _ _  ………………………………………………….. |

I hereby declare that I have fully informed this subject about the above-mentioned study. If during the research, information becomes available that could influence the consent of the subject, I will inform her in due time.

| **Researcher (or his representative)** |  |
| --- | --- |
| Name:  Date:  Signature: | …………………………………………………..  _ _ - _ _ - 2 0 _ _  ………………………………………………….. |
