# Supplementary material for: First trimester anomaly scan using virtual reality (VR FETUS study): study protocol for a randomized clinical trial
Source: BMC Pregnancy Childbirth. 2020 Sep 7;20:515. doi: 10.1186/s12884-020-03180-8 (PMC7487721; doi:10.1186/s12884-020-03180-8)
Supplement: Supplementary file 2 — Additional file 2. Information for future parents. [file 12884_2020_3180_MOESM2_ESM.docx]

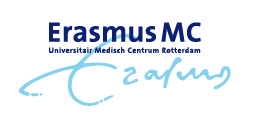


***Information for future parents***

***"The additional value of first trimester three-dimensional virtual reality ultrasound scan in obstetric care: a randomized study"***

***"The efficacy of three-dimensional virtual reality ultrasound during***

***the first three months of pregnancy”***

Dear Sir / Madam,

We would like to ask you for permission to use your child's data for a medical scientific study. During this study, we will investigate whether three-dimensional (3D) virtual reality (VR) ultrasound, a technique that can display depth, performed during the first three months of pregnancy can detect birth defects. Detailed information and a description of this scientific study can be found in the patient information leaflet of the Virtual Reality FETUS study.

In short, if the future mother chooses to participate in this study, she will be randomly assigned to one of the two study groups. In the intervention group, an extra (transvaginal) ultrasound scan will be made during the first three months of pregnancy with two-dimensional (2D) ultrasound and three-dimensional (3D) Virtual Reality techniques. In the other group, no extra ultrasound scan will be done during the first three months of pregnancy. In both groups, the future mother is offered normal care, i.e. an extensive structural ultrasound for birth defects around 20 weeks’ gestational age.

After giving birth, the researchers would like to request the details of the health of your child from the care provider(s) and authorities involved. This is necessary to properly assess the results of this study. The researchers therefore ask your permission to request the data of your child from the following authorities and persons:

- The Dutch Perinatal Registration Foundation (which stores coded data on the obstetrical outcomes of women who have given birth in the Netherlands).

- The Municipality / Statistics Netherlands (they have information on the family composition, whether you have moved and unexpected mortality has occurred).

- The Municipal Health Service (the Dutch National Public Health Institute collects information on the growth and development of children).

- The Dutch Foundation for Pharmaceutical Statistics (this collects data about the use of medication in the Netherlands).

- Your child's pharmacy.

- The pathologist (in the event of a miscarriage or stillbirth, we would like to obtain the results of the examination by the pathologist. This kind of examination will only be performed if you wish to make use of the option to perform this within the context of the standard care).

The general brochure, which has been handed over to the expectant mother, explains that the researcher collects data about participants and will treat it confidentially. These data include your age, ethnicity and education level. This means that a number of people may see the medical status and collected data of your child. These persons may use the data for the scientific study, but they may only make this data public without mentioning the name of your child or other personal data. Therefore, the identity of your child always remains secret. The researcher stores the data with a code. This means that instead of your child’s name, the research documents only contain a letter-digit code. Only the researcher maintains a list stating which letter-digit code belongs to which name.

Normally, only the treating physician and his / her team have access to your child's data. If your child participates in this study, more people may have access to the medical records and research data of your child. The persons who can review your child’s data are:

- the staff of the study team,

- the members of the review committee who approved the investigation,

- the authorized employees of the Health Care Inspectorate,

- persons designated by the investigators to monitor and supervise the investigation.

We are obliged to store the study data and ultrasound images of your child for 15 years after the end of the study. By participating in this study, you consent to storing this data for 15 years. If you do not want this, your child cannot participate in this study.

In addition, we might use your child's data and ultrasound images for other studies that are performed on child development and the detection of birth defects. These studies have the same objective as the study for which participation of your child is asked. Your child's data will not be used in a completely different study, with a completely different research subject or problem. It goes without saying that the confidentiality, as we have described above, always applies. Do you mind if we store the details of your child and use these for future research? If you don't want that, we will of course respect that. You can indicate your choice on the consent form. We may want to approach your child in the future for a follow-up study. You can indicate whether you consent to this on the consent form.

**Confidentiality of your child's data**

To protect your child's privacy the data will be coded. The name and other data that can directly identify your child are all anonymized. Tracing the data back to your child is only possible with a key of this code. The key of the code remains safely stored in the local research institution. The data sent to the client only contain the coded information, but not the name or other information that identify your child. In reports and publications of this study, data is not relatable to your child.

**Access your child’s data for review**

Some people may access all data of your child at the study site. Also to the decoded data. This is necessary in order to check whether the study has been carried out properly and reliably. Persons who have access to your child’s data for inspection are: the committee that monitors the safety of the investigation, a monitor that works for the sponsor of the investigation, national and international supervisory authorities (for example, the Health Care Inspectorate and Youth). They keep the details of your child secret. We ask you to give permission for this access.

**Data storage period**

Your child’s data should be kept at the study site for 15 years after the study ends.

**Retention and use of data for other research**

After this scientific study is completed, your child’s data may also be important for other scientific research in the area of ​​the further development of the ultrasound method. For this, your child’s data will be kept for 15 years after the end of the examination. You can indicate on the consent form whether you agree to this.

**Information about unexpected findings**

During this study, something may accidentally be found that is not important for the research but is important for your child. If this is important for the health of your child, you will be informed by the doctor or midwife of the Fetal Medicine Department. You can then discuss these findings with your midwife or physician. You can also give permission for sharing this information.

**Revoke permission**

You can always withdraw your permission to use your child’s personal data. This applies to this study and to the storage and use for future research. The study data collected until you withdraw your consent will still be used in the study.

**More information about your child’s rights when processing data**

For general information about the rights of your child when processing his / her personal data, you can consult the website of the Dutch Data Protection Authority.

If you have any questions about the rights of your child, please contact the person responsible for the processing of the personal data. This is the Erasmus MC for this study. See Appendix 1 for contact details.

If you have any questions or complaints about the processing of your child’s personal data, we recommend that you first contact the study team. You can also contact the Data Protection Officer of the institution, see appendix 1 for contact details. You can also contact the Dutch Data Protection Authority.

**Registration of the study**

Information about this study is also included in an overview of medical scientific studies, namely the Dutch Trial Register. It does not include any data that can be traced back to your child. After the study is finished, the website may show a summary of the results of this investigation. You will find this study under 'The use of three-dimensional virtual reality ultrasound during the first three months of pregnancy'.

**Do you have any questions?**

If you have any questions or complaints during the examination, we ask you to contact the treating physician of the expectant mother or a member of the study team: see appendix 1 for contact details.

If you are unsure about your child's participation, you can consult an independent doctor who is not involved in the study itself, but who is an expert in the field of this study. If you have questions, before or during the investigation, that you do not dare to propose to the investigators, you can contact the independent doctor.

If you are not satisfied with the investigation or treatment, you can contact the Complaints Office of the Erasmus MC University Medical Center.

If, after careful consideration, you have decided your child will participate in this scientific study, we ask you and your partner to sign and date the consent form together with the researcher.

Sincerely,

The study team

Dr. A.G.M.G.J. Mulders, gynecologist-obstetrician, Erasmus MC

Dr. M. Rousian, gynecologist in training, Erasmus MC

Drs. C.S. Pietersma, physician at the Fetal Medicine Department, Erasmus MC

**Appendix 1: Contact details**

Lead researcher:

Dr. A.G.M.G.J. Mulders, gynecologist-obstetrician tel. +3110 - 703 3492

Co-researcher:

Dr. M. Rousian, gynecologist in training tel. +3110 - 703 3492

Drs. C.S. Pietersma, physician Fetal Medicine tel. +316 - 2824 6981

Independent doctor:

Prof. dr. I.K.M. Reiss, neonatologist tel. +3110 - 703 60 77

Complaints Office of Erasmus MC University Medical Center:

tel. +3110 - 703 31 98

Data protection officer:

The Erasmus MC Data Protection Officer can be reached through the secretariat of the Legal Affairs Department: tel. +3110 - 703 49 86

Website of Dutch Data Protection Authority:

www.autoriteitpersoonsgegevens.nl

Website Dutch Trial Register – Virtual Reality FETUS study

<http://www.trialregister.nl/trialreg/admin/rctview.asp?TC=6309>

**Consent form future parents (copy parents)**

**"The added value of first trimester three-dimensional virtual reality ultrasound scan in obstetric care: a randomized study"**

**"The use of three-dimensional virtual reality ultrasound during the first three months of pregnancy”**

Study number: 

I confirm that I have read the information brochure about the study and understand the information. I have had the opportunity to ask additional questions. These questions were answered satisfactorily. I have had enough time to think about the participation of my child.

I know that the participation of my child is completely voluntary and that I can withdraw this consent at any time without motivation.

I authorize the viewing and retrieval of my child’s medical and research data by authorized employees of the research team, employees of the Health Care Inspectorate, members of the Medical Ethics Committee Erasmus MC and persons appointed by the researchers to check and aid the study.

I give permission to use my child’s data for the purposes as described in the information brochure.

I authorize the storage of my child’s research data and ultrasound images 15 years after the end of this research.

|  | **Yes** | **No** |
| --- | --- | --- |
| I give permission to use my child's research data and ultrasound images for other studies that are conducted on the child's development and detecting abnormalities. | □ | □ |
| I give permission to request information about my child from the Dutch Perinatal Register (which stores coded data on the obstetrics of women who have given birth in the Netherlands). | □ | □ |
| I give permission to request information about my child from the Municipality / Statistics Netherlands (they collect information on the family composition, whether you have moved and unexpected mortality has occurred). | □ | □ |
| I give permission to request information about my child from the Municipal Health Service (the Dutch National Public Health Institute collects information on the growth and development of children). | □ | □ |

|  | **Yes** | **No** |
| --- | --- | --- |
| I give permission to request information of my child from my child's pharmacy. | □ | □ |
| I give permission to request information about my child from the Dutch Foundation for Pharmaceutical Statistics (this collects data about the use of medication in the Netherlands) | □ | □ |
| I give permission to obtain the results of the examination by the pathologist in case of a miscarriage or stillbirth. (This kind of examination will only be performed if you wish to make use of the option to perform this within the context of standard care) | □ | □ |
| I give permission to approach my child in the future for a follow-up study. | □ | □ |

I give permission for my child’s participation in this study.

| **Expectant mother** |  |
| --- | --- |
| Name:  Date:  Signature: | …………………………………………………..  _ _ - _ _ - 2 0 _ _  ………………………………………………….. |

| **Expectant father** |  |
| --- | --- |
| Name:  Date:  Signature: | …………………………………………………..  _ _ - _ _ - 2 0 _ _  ………………………………………………….. |

I hereby declare that I have fully informed this subject about the above-mentioned study. If during the research information becomes available that could influence the consent of the subject, I will inform him / her in due time.

| **Researcher (or his representative)** |  |
| --- | --- |
| Name:  Date:  Signature: | …………………………………………………..  _ _ - _ _ - 2 0 _ _  ………………………………………………….. |

**Consent form future parents (copy researcher)**

**"The added value of first trimester three-dimensional virtual reality ultrasound scan in obstetric care: a randomized study"**

**"The use of three-dimensional virtual reality ultrasound during the first three months of pregnancy”**

Study number: 

I confirm that I have read the information brochure about the study and understand the information. I have had the opportunity to ask additional questions. These questions were answered satisfactorily. I have had enough time to think about the participation of my child.

I know that the participation of my child is completely voluntary and that I can withdraw this consent at any time without motivation.

I authorize the viewing and retrieval of my child’s medical and research data by authorized employees of the research team, employees of the Health Care Inspectorate, members of the Medical Ethics Committee Erasmus MC and persons appointed by the researchers to check and aid the study.

I give permission to use my child’s data for the purposes as described in the information brochure.

I authorize the storage of my child’s research data and ultrasound images 15 years after the end of this research.

|  | **Yes** | **No** |
| --- | --- | --- |
| I give permission to use my child's research data and ultrasound images for other studies that are conducted on the child's development and detecting abnormalities. | □ | □ |
| I give permission to request information about my child from the Dutch Perinatal Register (which stores coded data on the obstetrics of women who have given birth in the Netherlands). | □ | □ |
| I give permission to request information about my child from the Municipality / Statistics Netherlands (they collect information on the family composition, whether you have moved and unexpected mortality has occurred). | □ | □ |
| I give permission to request information about my child from the Municipal Health Service (the Dutch National Public Health Institute collects information on the growth and development of children). | □ | □ |

|  | **Yes** | **No** |
| --- | --- | --- |
| I give permission to request information of my child from my child's pharmacy. | □ | □ |
| I give permission to request information about my child from the Dutch Foundation for Pharmaceutical Statistics (this collects data about the use of medication in the Netherlands) | □ | □ |
| I give permission to obtain the results of the examination by the pathologist in case of a miscarriage or stillbirth. (This kind of examination will only be performed if you wish to make use of the option to perform this within the context of standard care) | □ | □ |
| I give permission to approach my child in the future for a follow-up study. | □ | □ |

I give permission for my child’s participation in this study.

| **Expectant mother** |  |
| --- | --- |
| Name:  Date:  Signature: | …………………………………………………..  _ _ - _ _ - 2 0 _ _  ………………………………………………….. |

| **Expectant father** |  |
| --- | --- |
| Name:  Date:  Signature: | …………………………………………………..  _ _ - _ _ - 2 0 _ _  ………………………………………………….. |

I hereby declare that I have fully informed this subject about the above-mentioned study. If during the research information becomes available that could influence the consent of the subject, I will inform him / her in due time.

| **Researcher (or his representative)** |  |
| --- | --- |
| Name:  Date:  Signature: | …………………………………………………..  _ _ - _ _ - 2 0 _ _  ………………………………………………….. |
