# Supplementary material for: First trimester anomaly scan using virtual reality (VR FETUS study): study protocol for a randomized clinical trial
Source: BMC Pregnancy Childbirth. 2020 Sep 7;20:515. doi: 10.1186/s12884-020-03180-8 (PMC7487721; doi:10.1186/s12884-020-03180-8)
Supplement: Supplementary file 3 — Additional file 3. Checklist structural ultrasound examination 1st trimester. [file 12884_2020_3180_MOESM3_ESM.docx]

**Supplement 1: Checklist structural ultrasound examination 1^st^ trimester**

**Fetal biometry / focus Structural Examination**

**Head**

| BPD, HC; ventricles optional  TCD; 3^rd^ ventricle optional  Intracranial translucency (IT) | Symmetry of choroid plexus, skull, falx cerebri |
| --- | --- |

**Face and neck**

| Nuchal translucency (NT), nasal bone  IOD/EOD  Lips  Mandible | Normal Profile, cranial bones  Eyes with lens  Intact lips  Intact mandible |
| --- | --- |

**Spine**

| Midsagittal CRL  Coronal spine | Vertebrae, intact skin  Vertebrae, intact skin |
| --- | --- |

**Chest**

| Lungs and heart  Diaphragm | Symmetrical lung fields, cardiac axis  Stomach and heart visible |
| --- | --- |

**Heart**

| Biventricular heart without color Doppler  Biventricular heart with color Doppler  Left outflow tract with color Doppler  Right outflow tract with color Doppler  3-vessel view with color Doppler  Trachea view with color Doppler  Tricuspid valve with pulsed wave or power Doppler | Two ventricles  Two ventricles  Crossing of the great arteries  Pulmonary artery, aorta and superior v cava  V-sign  Valve regurgitation |
| --- | --- |

**Abdomen**

| AC  Abdominal wall  Ductus venosus  Kidney length; renal pyelum optional  Bladder diameter | Stomach present in upper left quadrant  Normal cord insertion, no umbilical defects  Ductus venosus PI  Normal kidney position and appearance |
| --- | --- |

**Extremities**

| Femur  Left leg and foot  Right leg and foot  Left arm and hand  Right arm and hand | Femur length  Three segments  Three segments  Three segments  Three segments |
| --- | --- |

**Other**

| Three-vessel cord in color Doppler  Placenta-cervix  Fetal sex optional | Two umbilical arteries  Size and texture of the placenta |
| --- | --- |

**3D Volumes** Recordings Suggestion angle Degrees

| Fetus  Head  Extremities  Complete pregnancy  Abdominal wall and umbilical cord with color Doppler  STIC (heart) without color Doppler  STIC with color Doppler | 2 (perpendicular planes)  2 (perpendicular planes)  2 (separate arms and legs)  2 (perpendicular planes)  2  3  3 | 90-100  50-60  60  120  30  15-20  15-20 |
| --- | --- | --- |
